# Spatial distribution and regional difference of carbon emissions efficiency of industrial energy in China

**DOI:** 10.1038/s41598-021-98225-z

**Published:** 2021-09-30

**Authors:** Fang Liu, Lu Tang, Kaicheng Liao, Lijuan Ruan, Pingsheng Liu

**Affiliations:** 1 Business School, Jiangsu University of Technology, Changzhou, 213001 China; 2 Business School, Jishou University, Jishou, 416000 China; 3 School of Economics and Management, Tongji University, Shanghai, 200092 China; 4 Shiliang Law School, Changzhou University, Changzhou, 213164 China; 5 School of Finance and Commerce, Zhongshan Torch Polytechnic, Zhongshan, 528436 China

**Keywords:** Environmental sciences, Environmental social sciences

## Abstract

The three-stage super-efficiency slack-based measure and data envelopment analysis (SBM-DEA) model with undesirable outputs is used to calculate carbon emissions efficiency of industrial energy (CEEIE) of 30 provinces in China from 2000 to 2017. Then ArcGIS software is used to illustrate the spatial distribution of CEEIE, and Dagum Gini ratio is calculated to decompose the regional difference. The results show that the spatial distribution of CEEIE changes from disorder to order and provinces characterized with high or low CEEIE cluster in space over time. The total Dagum Gini coefficient indicates that the interprovincial difference in CEEIE across China is gradually expanding, which is mainly induced by the difference between regions. Our findings attach more importance to interregional integration policies for carbon emissions reduction in China.

## Introduction

The Covid-19 pandemic resulted in the largest-ever decline in global emissions, which indicated that global energy-related CO_2_ emissions fell by 5.8% in 2020. However, as the only major economy to record an increase in annual CO_2_ emissions in 2020, China’s emissions growth slows by just one percentage point compared with its average rate over the 2015–2019 period. The latest annual figures indicate that the country’s overall CO_2_ emissions in 2020 were 0.8% (or 75 Mt CO2) above the levels assessed at the end of 2019^1^. The country has entered a new normal in which development mode has changed largely, which has large impacts on carbon emissions^2^. In addition, China has committed to achieve carbon peak by 2030 and carbon neutrality by 2060. Ensuring economic growth and the realization of the two targets will be extremely challenging for Chinese government. As we know, industry always plays an important role in the economy and has been the dominant energy consumer and carbon emitter. In 2020, Chinese industry contributed 37.8% increments of GDP (National Bureau of Statistics of China 2021). Meanwhile, the proportion of energy consumption in the industrial sector is always higher than 65% and the carbon dioxide emissions by industry accounts for a share of over 70%. Therefore, reducing the carbon emissions of industrial energy consumption is a major challenge for China, which requires that the government pays more attention to carbon emissions efficiency of industrial energy (CEEIE).

As for how to implement the policies to curb the carbon emissions of industrial energy, first we should know the situation of carbon emissions efficiency. Kaya and Yokobori^3^ and Sun^4^ believe that the carbon emissions per unit of GDP can measure carbon emissions efficiency. Mielnik and Goldemberg^5^ use the ratio of carbon emissions to energy consumption to measure carbon emissions efficiency. As direct measurement methods, these kinds of a single indicator have noticeable limitations, because it does not take a wide range of important external factors into consideration (Wang et al.^6^; Cheng et al.^7^). Although there are many methods for measuring carbon emissions efficiency from total factors perspective, data envelopment analysis (DEA, an efficiency evaluation method that provides a comprehensive evaluation of the relative effectiveness of similar decision-making units) has been the most widely used method (Ramanathan^8^; Zhou et al.^9^; Guo et al.^10^; Meng et al.^11^; Zhou et al.^12^; Cheng et al.^13^).

Second, it is vital to pay attention to regional difference of carbon emissions efficiency because of its variety across many regions in some countries, especially in those with many provincial regions like China. Guo et al.^10^ use the DEA method to evaluate and compare the carbon emissions performance of 29 Chinese administrative regions at provincial level. Instead of DEA, Dong et al.^14^ use a stochastic frontier analysis (SFA) method to measure and compare the carbon emissions efficiency of some provinces in China. Meng et al.^11^ also use DEA to evaluate the variation of carbon emissions efficiency across 31 provinces in China. In China, developed provinces tend to have higher carbon emissions efficiency compared with less developed provinces^15^. Some research focus on carbon emissions efficiency differentiation and variations among cities other than provinces. Similar to the findings of^15^, Wang and Wei^16^ find that developed cities have high carbon emissions efficiency because of strong economic development capabilities, while the carbon emissions efficiency of less developed cities are low because of weak economic development capabilities in China. Cheng et al.^7^ find that eastern China has the largest carbon emissions efficiency of provincial industrial sectors, and western China has the lowest.

Third, carbon emissions efficiency might be spatially correlated because environmental governance is spatially dependent^17^. Spatial heterogeneity and spatial spillover effect of carbon emissions efficiency have been identified in many studies. Liu et al.^18^ use the k-means cluster method to analyze carbon dioxide emission efficiency of China and identity five groups of energy consumption structure. Zhang et al.^19^ find the spatial spillover effect of CO_2_ emissions efficiency in China. The findings of Yan et al.^15^ show that the power sectors of eastern regions have relatively high carbon emissions efficiency and tend to have a positive spillover effect on the neighboring provinces. Wang et al.^20^ find that CO_2_ emissions of China have shown a stable spatial agglomeration effect from global and local perspectives because of the similarity and connectivity of spatial units. Provinces and cities with high carbon emissions efficiency in China cluster in mid-east coastal regions^21^, while provinces and cities with low carbon emissions efficiency cluster in northern part of China^22^. Since the clustering effect is found to be quite obvious in China, some other researchers even initially partitioned clustering areas based on carbon emissions efficiency by using the quantitative methodology. For example, Ding et al.^23^ find that the eastern zone is the most advanced in carbon emission efficiency in China.

Although the studies mentioned above can obtain the three key information of carbon emissions efficiency, i.e., the efficiency value, regional difference and spatial characteristics, at least three aspects should be improved. First, the traditional DEA model^24^ may overestimate the efficiency value of decision-making unit (DMU) if there are nonzero slack variables. Slack based measure (SBM) DEA^25^ can solve the problem resulting from slack variables, while super efficiency-SBM can evaluate the efficiency of DMU if there are more than one efficient DMU. But when these methods are used to measure carbon emissions efficiency, the environmental difference among regions should be ignored. Combining traditional DEA with SFA, the three-stage DEA method can eliminate the impact of environmental factors and random errors on efficiency value^26^. In addition, carbon emission, as an output, is undesirable from the economic perspective. Therefore, we argue that it is more reasonable to use a three-stage super-efficiency SBM-DEA method with undesirable output to measure carbon emissions efficiency. Second, the findings of regional difference of carbon emissions efficiency is descriptive, and cannot identify the key factors inducing the difference and provide more information for policymaker. While the Dagum Gini ratio initiated by Dagum^27^ can obtain the true difference of carbon emissions efficiency among regions, and also can get the source of the difference. Third, many literatures have studies carbon emissions efficiency from the perspective of nation, specific sub-sectors or specific regions. However, few studies focus on industrial energy consumption, which is highly correlated with carbon emissions efficiency. We thus attach more importance to CEEIE and target the effective solutions to reduce carbon emissions of industrial energy sectors.

Specifically, in order to avoid the lacks of traditional DEA model, we combine the three-stage DEA method with the super-SBM to construct an input–output framework and evaluate CEEIE. Then we illustrate the spatial distribution of CEEIE to discern the evolution trend over time. Besides, Dagum Gini ratio is used to decompose the regional difference of CEEIE, and then the sources of regional difference and spatial pattern of regional CEEIE are analyzed. Our findings can help the government to identify the spatial disequilibrium characteristics of CEEIE and are beneficial for balanced policy making of carbon emissions reduction from the regional perspective.

## Methodology and data sources

### The method for measuring CEEIE

In this paper, we use the three-stage super-efficiency SBM-DEA method^17^ with undesirable output to measure the CEEIE of the 30 provinces in China. It is necessary to point out that the carbon emission is regarded as an undesirable output in the process of the calculation of CEEIE. We divide the outputs into desirable output and undesirable output in terms of the classification method proposed by Wang and Luo^28^. The desirable output meets the expectation and is beneficial for society, but the undesirable output is just on the opposite. In addition, there are 34 provinces in China. For the reason of the lack of data, Tibet, Macau, Hong Kong and Taiwan are excluded from the sample. Therefore, the 30 provinces are Beijing, Tianjin, Hebei, Liaoning, Shanghai, Jiangsu, Zhejiang, Fujian, Shandong, Guangdong, Guangxi, Hainan, Shanxi, Inner Mongolia, Jilin, Heilongjiang, Anhui, Jiangxi, Henan, Hubei, Hunan, Chongqing, Sichuan, Guizhou, Yunnan, Shaanxi, Gansu, Qinghai, Ningxia and Xinjiang. In the first stage, the super-efficiency SBM-DEA model is used to calculate the initial efficiency value and slack value of input/output for each DMU. Since there are zero values in our input data, we use the input-oriented super-efficiency SBM-DEA model

1 $$ \begin{aligned} & {\text{min}}\rho = \frac{{\frac{1}{m}\mathop \sum \nolimits_{i = 1}^{m} {\raise0.7ex\hbox{${\overline{x}_{i} }$} \!\mathord{\left/ {\vphantom {{\overline{x}_{i} } {x_{ik} }}}\right.\kern-\nulldelimiterspace} \!\lower0.7ex\hbox{${x_{ik} }$}}}}{{\frac{1}{s}\mathop \sum \nolimits_{r = 1}^{s} {\raise0.7ex\hbox{${\overline{y}_{r} }$} \!\mathord{\left/ {\vphantom {{\overline{y}_{r} } {y_{rk} }}}\right.\kern-\nulldelimiterspace} \!\lower0.7ex\hbox{${y_{rk} }$}}}} \\ & {\text{s}}.{\text{t}}.{ }\mathop \sum \limits_{j = 1, j \ne k}^{n} \lambda_{j} x_{j} < \overline{x}, \mathop \sum \limits_{j = 1, j \ne k}^{n} \lambda_{j} y_{j} \le \overline{y} \\ & x_{ik} = \mathop \sum \limits_{i = 1}^{n} \lambda_{j} x_{ij} + s^{ - } , y_{rk} = \mathop \sum \limits_{i = 1}^{n} \lambda_{j} y_{rj} - s^{ + } \\ & \overline{x} \ge x_{k} , \overline{y} \le y_{k} \\ & \mathop \sum \limits_{j = 1,j \ne k}^{n} \lambda_{j} = 1,\overline{y} \ge 0,\lambda \ge 0,s^{ - } \ge 0,s^{ + } \ge 0 \\ \end{aligned} $$

where ${ }\rho { }$ is the efficiency value, and *m*, *s* and *n* are the number of input indicators, output indicators and DMU. ${s}^{-}$ is the slack variable of the input, ${s}^{+}$ is the undesirable output slack variable. *x* and *y* are matrixes that compose of the input and output of all DMU, and $\overline{x }$ and $\overline{y  }$ are the reference point of the decision variable. *λ* is the weight vector. $\rho <1$ indicates that the DMU is inefficient, while $\rho >1$ indicates that DMU is in an active state.

In the second stage, SFA is used to decompose the slack values obtained in the first stage. SFA (Aigner et al.^29^; Meeusen and van den Broeck^30^) is regression-based, and so has the virtue of being capable of isolating managerial inefficiency from both environmental effects and statistical noise, although it has the drawback of doing so within a parametric framework. Fried et al.^26^ point out that the traditional DEA model cannot identify the inefficiency caused by managerial factors, environmental factors and random factors. Therefore, the way to solve this problem is to use SFA model at the second stage to screen out the impact of external environmental factors and random errors, and then the redundant input values are entirely caused by managerial inefficiency. The SFA regression model is

2 $${S}_{in}={f}_{i}\left({Z}_{i},{\beta }_{n}\right)+{v}_{ni}+{\mu }_{ni}(i=\mathrm{1,2},\dots ,m;\quad n=\mathrm{1,2},\dots ,N)$$

where ${S}_{ni}$ is the redundancy of the *n*-th input of the *i*-th DMU, ${Z}_{i}$ is the environment variable vector and ${\beta }_{n}$ is the coefficient vector to be estimated. ${\varepsilon }_{ni}{=v}_{ni}+{\mu }_{ni}$ represents the mixed error, in which ${v}_{ni}$ is the random error and ${\mu }_{ni}$ is the managerial inefficiency, and the two terms are independent. *v* ~ *N (0,*${\sigma }_{v}^{2}$*)* means that *v* follows the normal distribution, while *μ* ~ ${N}^{+}$ (*0, *${\sigma }_{\mu }^{2}$) means that *μ* follows the truncated normal distribution.$\gamma ={\sigma }_{v}^{2}/({\sigma }_{v}^{2}+{\sigma }_{\mu }^{2})$ is the ratio of management inefficiency variance to the total variance. When the $\gamma$ converges to 1, the managerial factor has the whole influence. When the $\gamma$ converges to 0, ${\mu }_{ni}$ equals 0 and the random model becomes a deterministic model, which can be estimated by ordinary least square. In order to adjust all the DMU to the same external environment and separate the random error from the mixed error terms, Frontier 4.1 is used to implement maximum likelihood estimation to get the estimation of ${\beta }_{n}$, ${\sigma }^{2}$ and $\gamma$. Then the method proposed by Jondrow et al.^31^ can be used to obtain the management inefficiency estimation.

3 $$\widehat{E}\left[{\mu }_{ni}|{v}_{ni}+{\mu }_{ni}\right]=\frac{\gamma \sigma }{1+{\gamma }^{2}}\left(\frac{\varphi (\gamma {e}_{n})}{\varnothing (\gamma {e}_{n})}+\gamma {e}_{n}\right)$$

where $\varnothing$ and $\varphi$ are the distribution function and density function of the standard normal distribution, and ${e}_{n}$ is the error of maximum likelihood estimation. The estimation of ${v}_{ni}$ is

4 $$\widehat{E}\left[{v}_{ni}|{v}_{ni}+{v}_{ni}\right]={S}_{ni}-f\left({Z}_{i},{\beta }_{n}\right)-\widehat{E}\left[{\mu }_{ni}|{v}_{ni}+{\mu }_{ni}\right]$$

And then the input data are adjusted by Eq. (5).

5 $$ \begin{aligned} & X_{ni}^{^{\prime}} = X_{ni} + \left\{ {max\left[ {f\left( {Z_{i} ;\hat{\beta }_{n} } \right)} \right] - f\left( {Z_{i} ;\hat{\beta }_{n} } \right)} \right\} + \left[ {max\left( {v_{ni} } \right) - v_{ni} } \right] \\ & \quad \left( {i = 1,2, \ldots ,I;\;\;n = 1,2, \ldots ,N} \right) \\ \end{aligned} $$

where ${X}_{ni}$ is the original input data, and ${X}_{ni }^{\prime}$ is the adjusted input data. $\left\{max\left[f\left({Z}_{i};{\widehat{\beta }}_{n}\right)\right]-f\left({Z}_{i};{\widehat{\beta }}_{n}\right)\right\}$ means that all DMUs are adjusted by the same environmental condition, and $\left[max\left({v}_{ni}\right)-{v}_{ni}\right]$ means that all DMUs are adjusted by the same random error.

In the third stage, ${X}_{ni}$ is replaced by ${X}_{ni }^{\prime}$, and then we can use the model (1) again to get CEEIE, which has excluded the impact of the environmental and random factors.

### The method for decomposing regional difference of CEEIE

We use the Dagum Gini ratio method described by Peng et al.^17^ to decompose the regional difference of CEEIE. Dagum Gini ratio is defined as

6 $$ G{ = }{{\sum\limits_{i = 1}^{K} {\sum\limits_{j = 1}^{K} {\sum\limits_{h = 1}^{{n_{i} }} {\sum\limits_{r = 1}^{{n_{j} }} {\left| {y_{ih} - y_{jr} } \right|} } } } } \mathord{\left/ {\vphantom {{\sum\limits_{i = 1}^{K} {\sum\limits_{j = 1}^{K} {\sum\limits_{h = 1}^{{n_{i} }} {\sum\limits_{r = 1}^{{n_{j} }} {\left| {y_{ih} - y_{jr} } \right|} } } } } {2n^{2} \mu }}} \right. \kern-\nulldelimiterspace} {2n^{2} \mu }} $$

In the formula (6), *G* is the total Gini ratio, which measures the total difference of CEEIE between provinces. *K* is the number of regions, including northern coast, eastern coast, southern coast, northeast, middle Yellow River, middle Yangtze River, southwest and northwest region in this paper. The eight regions are classified according the report, Strategy and Policy of Regional Coordinated Development, published by Development Research Center of the State Council of China in 2005. And northern coast region includes Beijing, Tianjin, Hebei and Shandong, eastern coast region includes Shanghai, Jiangsu and Zhejiang, southern coast region includes Fujian, Guangdong and Hainan, northeast region includes Liaoning, Jilin and Heilongjiang, middle Yangtze River region includes Anhui, Jiangxi, Hubei and Hunan, middle Yellow River region includes Henan, Shanxi, Inner Mongolia and Shaanxi, southwest region includes Chongqing, Sichuan, Guizhou, Yunnan and Guangxi, and northwest region includes Gansu, Qinghai, Ningxia and Xinjiang. *y*_*ih*_ and *y*_*jr*_ are the CEEIE of provinces in the *i*-th and the *j*-th region, respectively, and *i* = 1,2 , …, *K*; *j* = 1, 2,…, *K*. *μ* is the average of CEEIE of all provinces, *n* is the number of all provinces, and *n*_*i*_ and *n*_*j*_ are the number of provinces in the *i*-th and the *j*-th region, respectively.

Like the method of Dagum, the total Gini ratio can be decomposed as follows

7 $$ G = G_{w} + G_{rb} + G_{t} $$

with

8 $$ G_{w} { = }\sum\limits_{i = 1}^{K} {\lambda_{i} s_{i} G_{ii} } $$

measures the contribution of the difference of CEEIE within region to the total Gini coefficient *G*;

9 $$ G_{rb} { = }\sum\limits_{i = 2}^{K} {\sum\limits_{j = 1}^{i - 1} {\left( {\lambda_{j} s_{i} + \lambda_{i} s_{j} } \right)} } G_{ij} D_{ij} $$

measures the net contribution of the extended difference of CEEIE between regions to the total Gini coefficient *G*;

10 $$ G_{t} { = }\sum\limits_{i = 2}^{K} {\sum\limits_{j = 1}^{i = 1} {\left( {\lambda_{j} s_{i} + \lambda_{i} s_{j} } \right)} } G_{ij} (1 - D_{ij} ) $$

measures the contribution of the transvariation intensity between regions to the total Gini coefficient *G*. *λ*_*i*_ = *n*_*i*_*/n* and *s*_*i*_ = *λ*_*i*_*μ*_*i*_*/μ*, *μ*_*i*_ and *μ*_*j*_ are the average of CEEIE of the *i*-th and the *j*-th region.

In Eq. (10), *D*_*ij*_ = (*d*_*ij*_ − *p*_*ij*_)/(*d*_*ij*_ + *p*_*ij*_) is the relative economic affluence between the *i*-th and the *j*-th region, and the gross economic affluence *d*_*ij*_ between the *i*-th and the *j*-th region, such as *μ*_*i*_** > ***μ*_*j*_, is

11 $$ d_{ij} = \int_{0}^{\infty } {\int_{0}^{y} {\left( {y - x} \right)} } f_{i} (x)dxf_{j} (y)dy $$

where *f*_*i*_(*y*) and *f*_*j*_(*y*) are the probability density function of the *i*-th and the *j*-th region. *d*_*ij*_ is by definition the weighted average of the CEEIE difference *y*_*ih*_*–y*_*jr*_ for all CEEIE *y*_*ih*_ of the members belonging to the *i*-th region with CEEIE greater than *y*_*jr*_ of the members belonging to the *j*-th region, such that, *μ*_*i*_** > ***μ*_*j*_.

*p*_*ij*_ is the first-order moment of transvariation between the *i*-th and the *j*-th region, such that *μ*_*i*_** > ***μ*_*j*_, is

12 $$ p_{ij} = \int_{0}^{\infty } {\int_{0}^{y} {\left( {y - x} \right)} } f_{i} (x)dxf_{j} (y)dy $$

By definition *p*_*ij*_ is the weighted average of the CEEIE difference *y*_*ih*_** − ***y*_*jr*_ for all pairs of provinces, one taken from the *i*-th and the other from the *j*-th region, such that *y*_*ih*_* > y*_*jr*_ and *μ*_*i*_* > μ*_*j*_. The word transvariation stands to the fact that the differences in CEEIE considered are of opposite sign than the difference in means of their corresponding region.

*G*_*ii*_ is the Gini ratio within region and *G*_*ij*_ is the Gini ratio between regions*,* i.e.,

13 $$ G_{ii} { = }{{\sum\limits_{h = 1}^{{n_{i} }} {\sum\limits_{r = 1}^{{n_{j} }} {\left| {y_{ih} - y_{jr} } \right|} } } \mathord{\left/ {\vphantom {{\sum\limits_{h = 1}^{{n_{i} }} {\sum\limits_{r = 1}^{{n_{j} }} {\left| {y_{ih} - y_{jr} } \right|} } } {2n_{i}^{2} \mu_{i} }}} \right. \kern-\nulldelimiterspace} {2n_{i}^{2} \mu_{i} }} $$

14 $$ G_{ij} { = }{{\sum\limits_{h = 1}^{{n_{i} }} {\sum\limits_{r = 1}^{{n_{j} }} {\left| {y_{ih} - y_{jr} } \right|} } } \mathord{\left/ {\vphantom {{\sum\limits_{h = 1}^{{n_{i} }} {\sum\limits_{r = 1}^{{n_{j} }} {\left| {y_{ih} - y_{jr} } \right|} } } {n_{i} n_{j} (\mu_{i} + \mu_{j} )}}} \right. \kern-\nulldelimiterspace} {n_{i} n_{j} (\mu_{i} + \mu_{j} )}} $$

### Indicators and data sources

It’s vital to construct a comprehensive and objective input–output indicator system for accurately measuring the CEEIE in China. According to the relevant literatures and the availability of data, the input indicators are composed of labor, capital stock (calculated with the method proposed by Shan^32^) and energy consumption (the sum of all kinds of energy consumed by material production and non-material production sectors). We divide the output into desirable output (gross value of industrial output, the total amount of industrial products sold or available for sale produced by industrial enterprises in the form of currency) and undesirable output (carbon dioxide emissions, calculated according to the Guidelines for National Greenhouse Gas Inventories (Intergovernmental Panel on Climate Change (IPCC) 2006)). We use the following equation to calculation the CO_2_ emissions from industrial energy combustion on the basis of China’s provincial energy

15 $$ CO_{2} = \sum\limits_{i = 1}^{8} {E_{i} \times NCV_{i} \times CEC_{i} } $$

where *E* refers to the amount of energy consumption from different fuel types (emission factors), *NCV* refers to the net calorific value of different fuel types and *CEC* refers to the carbon emissions coefficient. Although Mi et al.^33^ attach more importance to the uncertainties in emission factors, we use the specific information in Table 1 for the formula (15) because of the limit of data.

**Table 1:** Fuel types, net calorific value and carbon emissions coefficient.

Fuel types	Coal	Coke	Crude oil	Gasoline	Kerosene	Diesel oil	Fuel oil	Natural gas
*NCV* (kj/kg(m^3^))	20,908	283,435	41,816	43,070	43,070	46,252	41,816	38,931
*CEC* (kg CO_2_/TJ)	95,333	107,000	73,300	70,000	71,500	74,100	77,400	56,100

The data of *NCV* is from China Energy Statistical Yearbook and the data of *CEC* is from IPCC 2006.

Many studies have found that carbon emissions efficiency is affected by many environmental factors. For example, technological progress can induce high carbon emissions efficiency^12^, and the level of economic development, economic capability, and energy structure also can lead to different carbon emissions efficiency^34^. We thus choose six environmental indicators from the perspective of economy, energy and institution.

The environmental indicators related to economy compose of GDP per capita (for measuring economic development) and the ratio of GDP of tertiary industry to GDP (for measuring industrial structure). On one hand, economic development means greater energy consumption and more carbon emissions. One other hand, infrastructure, energy utilization and pollutant treatment capacity will be improved with economic development. Therefore, the impact of economic development on CEEIE is not clear. As for industrial structure, it can affect CEEIE through energy consumption and energy intensity. For example, the optimization of industrial structure can promote the development of low-carbon industry to induce carbon dioxide emissions. The environmental indicator related to energy is the ratio of coal consumption to total energy consumption (for measuring energy consumption structure). According to the statistics of IPCC (2006), the carbon emissions per unit coal consumption is 1.33 times that of oil and 1.73 times that of natural gas. Therefore, a high coal consumption rate means low carbon emissions efficiency or a poor energy consumption structure. The environmental indicators related to institution compose of the ratio of government investment in environmental governance to GDP (for government environmental governance), the ratio of R&D expenditure to GDP (for measuring technological innovation ability) and the ratio of total import and export to GDP (for measuring the degree of opening up). The expenditure in government environmental governance and technological innovation can curb energy consumption of enterprises and promote the production and use of clean energy. With the increasing degree of opening up, the international organizations require China to make more contribution to carbon reduction. Besides, the effect of technology spillover brought by opening up should enhance energy efficiency.

The above-mentioned indicators are described in Table 2. All data in this paper are from China Statistical Yearbook, China Statistical Yearbook on Environment, China Energy Statistical Yearbook, China Labor Statistical Yearbook, Statistical Yearbook of the Chinese Investment in Fixed Assets, China Statistical Yearbook on Science and Technology, National Bureau of Statistics, and Statistical Yearbook and Bulletin of each province. Because of the lack of data, Tibet, Macau, Hong Kong and Taiwan are excluded from the sample, which consists of 30 provinces ultimately.

**Table 2:** Indicators for measuring CEEIE.

Categories	Description	Indicators	Unit	Data sources
Input	Labor input	Employment	Ten thousand people	China Labor Statistical Yearbook
	Capital input	Capital stock	Hundred million Yuan	Statistical Yearbook of the Chinese Investment in Fixed Assets, China Statistical Yearbook
	Energy input	Energy consumption	Ten thousand tons of standard coal	China Energy Statistical Yearbook
Output	Desirable output	Gross value of industrial output	Hundred million Yuan	China Statistical Yearbook
	Undesirable output	Carbon dioxide emissions	Ten thousand tons	Statistical Yearbook and Bulletin of each province, China Energy Statistical Yearbook, National Bureau of Statistics
Environment	Economic development	GDP per capita	Yuan	China Statistical Yearbook
	Industrial structure	The ratio of GDP of tertiary industry to GDP	%	China Statistical Yearbook
	Energy consumption structure	The ratio of coal consumption to energy consumption	%	China Energy Statistical Yearbook
	Government regulation	The ratio of government investment in environmental governance to GDP	%	China Statistical Yearbook on Environment
	Technological innovation	The ratio of R&D expenditure to GDP	%	China Statistical Yearbook
	Opening up	The ratio of total import and export to GDP	%	China Statistical Yearbook

## Results

### The situation of CEEIE

Table 3 reports the averages of CEEIE of China and the eight regions. From 2000 to 2019, the CEEIE of China is increasing significantly, and the average annual growth rate is 1.27%. Besides, the CEEIE of China ranges from 0.818 to 1.041, which implies a high level of carbon emissions efficiency. As for the regional CEEIE, northern coast region has the highest average annual growth rate (3.42%), while northeast region has the lowest (0.11%). In 2000, only the southern coast region has DEA effective CEEIE. But in 2019, there are five regions with DEA effective CEEIE and most of them (three out of five) cluster in coast. As we know, coastal regions in China have high level of economic development and advanced production technology, which means that they have greater abilities to reduce the carbon emissions.

**Table 3:** The averages of CEEIE of China and the eight regions from 2000 to 2019.

	China	Northern coast region	Eastern coast region	Southern coast region	Northeast region	Middle Yellow River region	Middle Yangtze River region	Southwest region	Northwest region
2000	0.818	0.687	0.814	1.440	0.780	0.813	0.743	0.858	0.546
2001	0.871	0.722	0.929	1.534	0.810	0.797	0.817	0.824	0.710
2002	0.885	0.786	0.859	1.165	0.920	0.861	0.895	0.917	0.740
2003	0.929	0.946	1.039	1.148	0.850	0.837	0.817	0.916	0.948
2004	0.814	0.937	0.955	1.231	0.737	0.624	0.663	0.772	0.722
2005	0.835	0.959	0.957	1.173	0.753	0.830	0.736	0.688	0.712
2006	0.890	1.026	0.960	1.249	0.759	0.905	0.797	0.864	0.642
2007	0.894	1.037	0.952	1.297	0.757	0.934	0.815	0.846	0.605
2008	0.914	1.063	0.942	1.394	0.757	0.962	0.805	0.845	0.648
2009	0.906	1.050	0.958	1.174	0.689	0.963	0.846	0.842	0.764
2010	0.921	1.052	0.983	1.427	0.776	0.978	0.936	0.761	0.599
2011	0.918	1.095	1.013	1.238	0.826	0.945	0.913	0.757	0.680
2012	0.932	1.106	0.982	1.522	0.735	0.966	0.963	0.747	0.591
2013	0.906	1.151	0.932	1.360	0.716	0.901	0.931	0.756	0.614
2014	0.897	1.153	0.972	1.407	0.799	0.827	0.802	0.817	0.543
2015	0.939	1.093	0.935	1.525	0.879	0.831	0.913	0.927	0.545
2016	0.947	1.161	1.010	1.610	0.738	0.835	0.865	0.922	0.567
2017	0.931	1.221	1.007	1.405	0.748	0.749	0.907	0.932	0.571
2018	0.999	1.273	1.086	1.547	0.776	0.806	0.961	1.005	0.637
2019	1.041	1.301	1.104	1.605	0.796	0.844	1.030	1.069	0.664
Average annual growth rate (%)	1.27	3.42	1.62	0.57	0.11	0.20	1.73	1.17	1.04

### The spatial distribution of CEEIE

**Figure 1:**
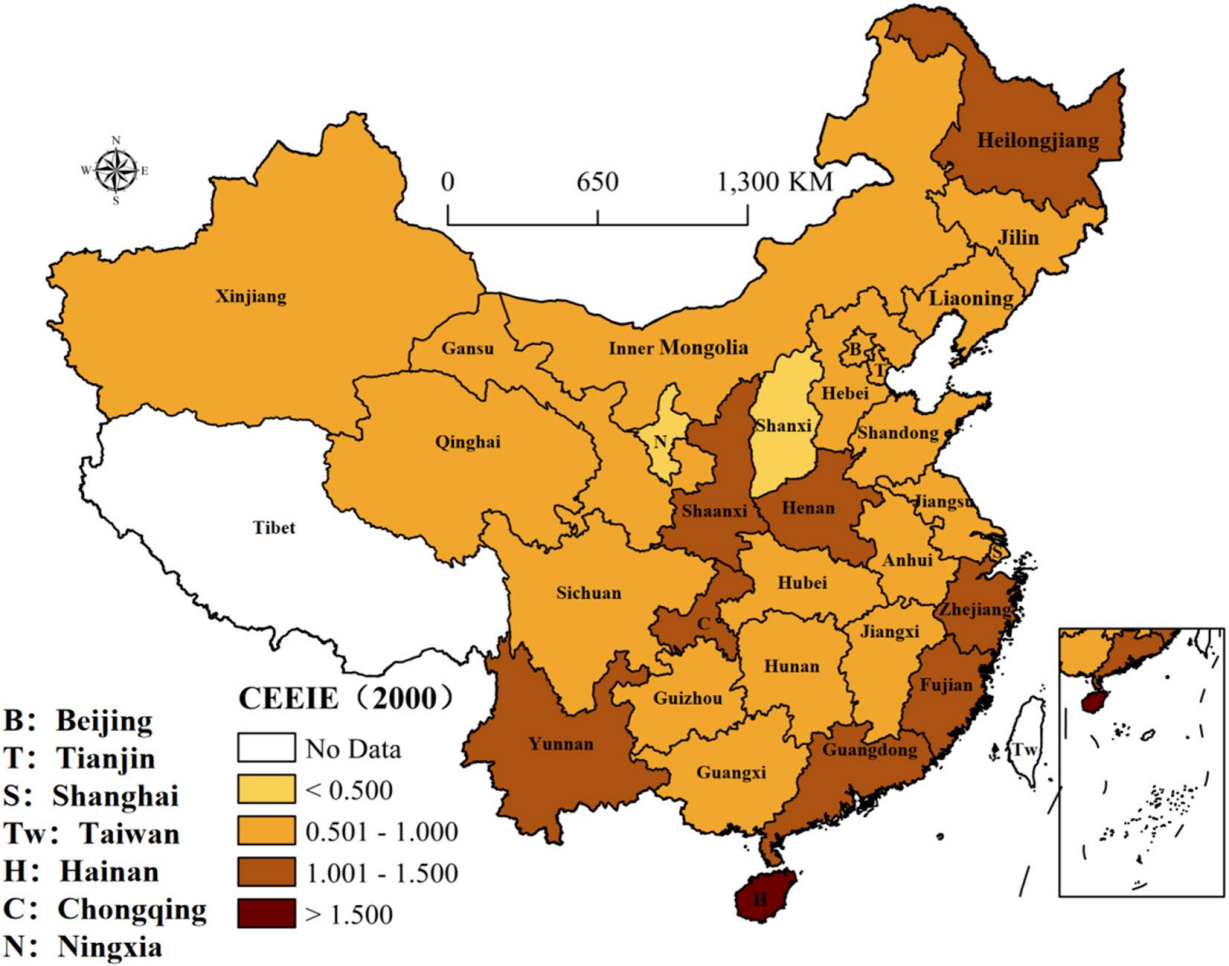
The distribution of CEEIE in 2000 (prepared by KL in ArcGIS Pro, https://www.esri.com/zh-cn/arcgis/products/arcgis-pro/resources).

**Figure 2:**
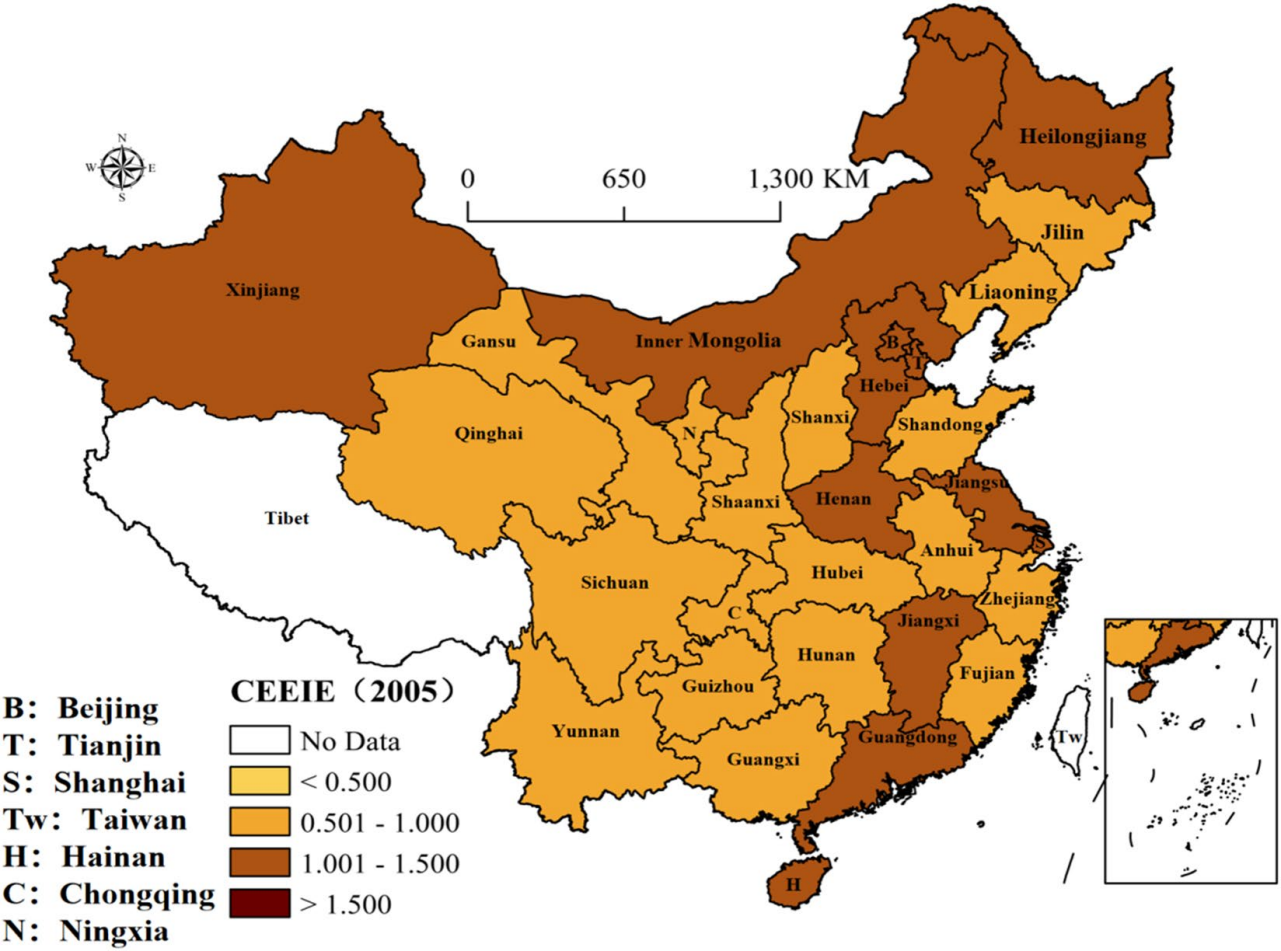
The distribution of CEEIE in 2005 (prepared by KL in ArcGIS Pro, https://www.esri.com/zh-cn/arcgis/products/arcgis-pro/resources).

**Figure 3:**
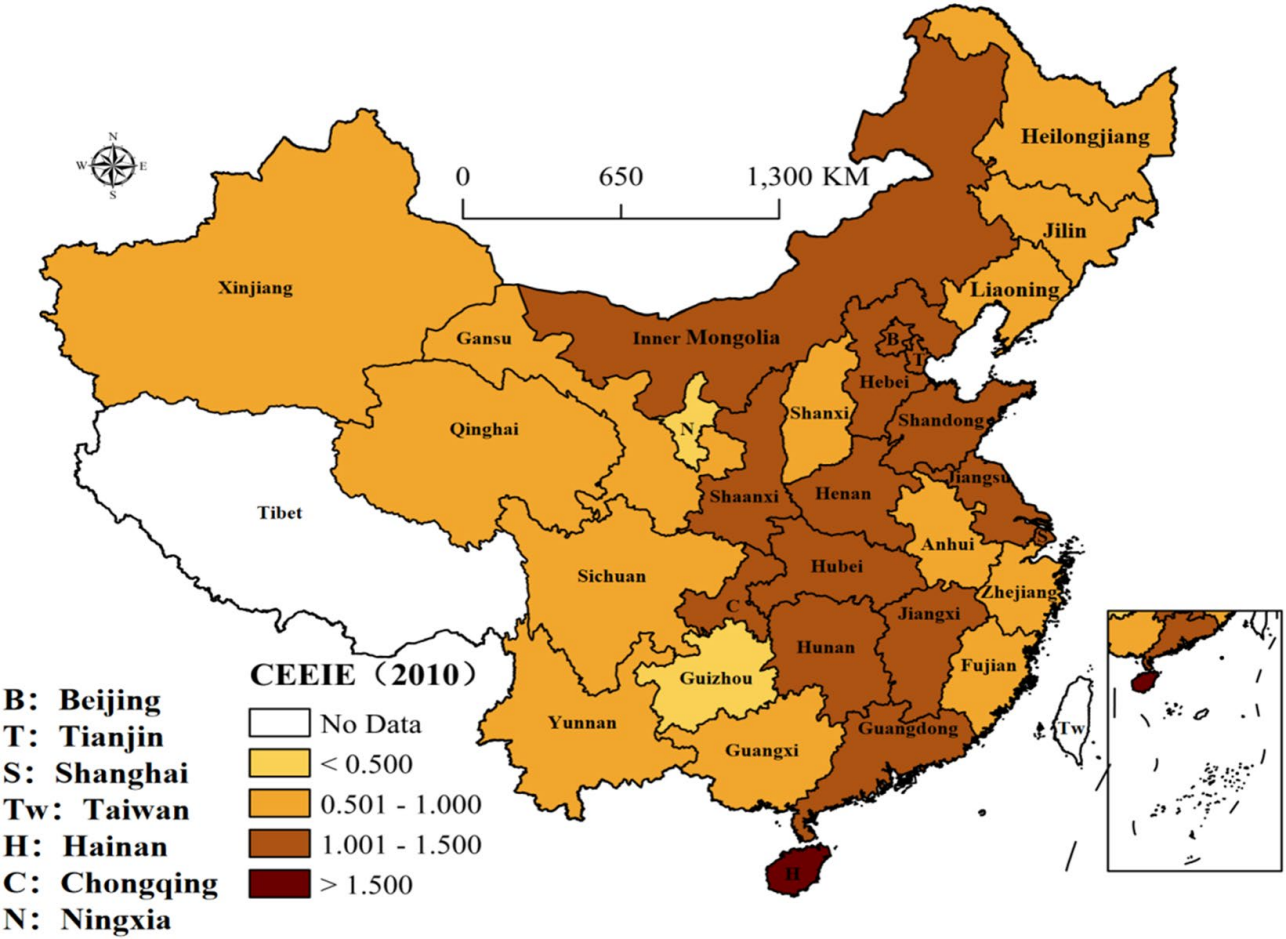
The distribution of CEEIE in 2010 (prepared by KL in ArcGIS Pro, https://www.esri.com/zh-cn/arcgis/products/arcgis-pro/resources).

**Figure 4:**
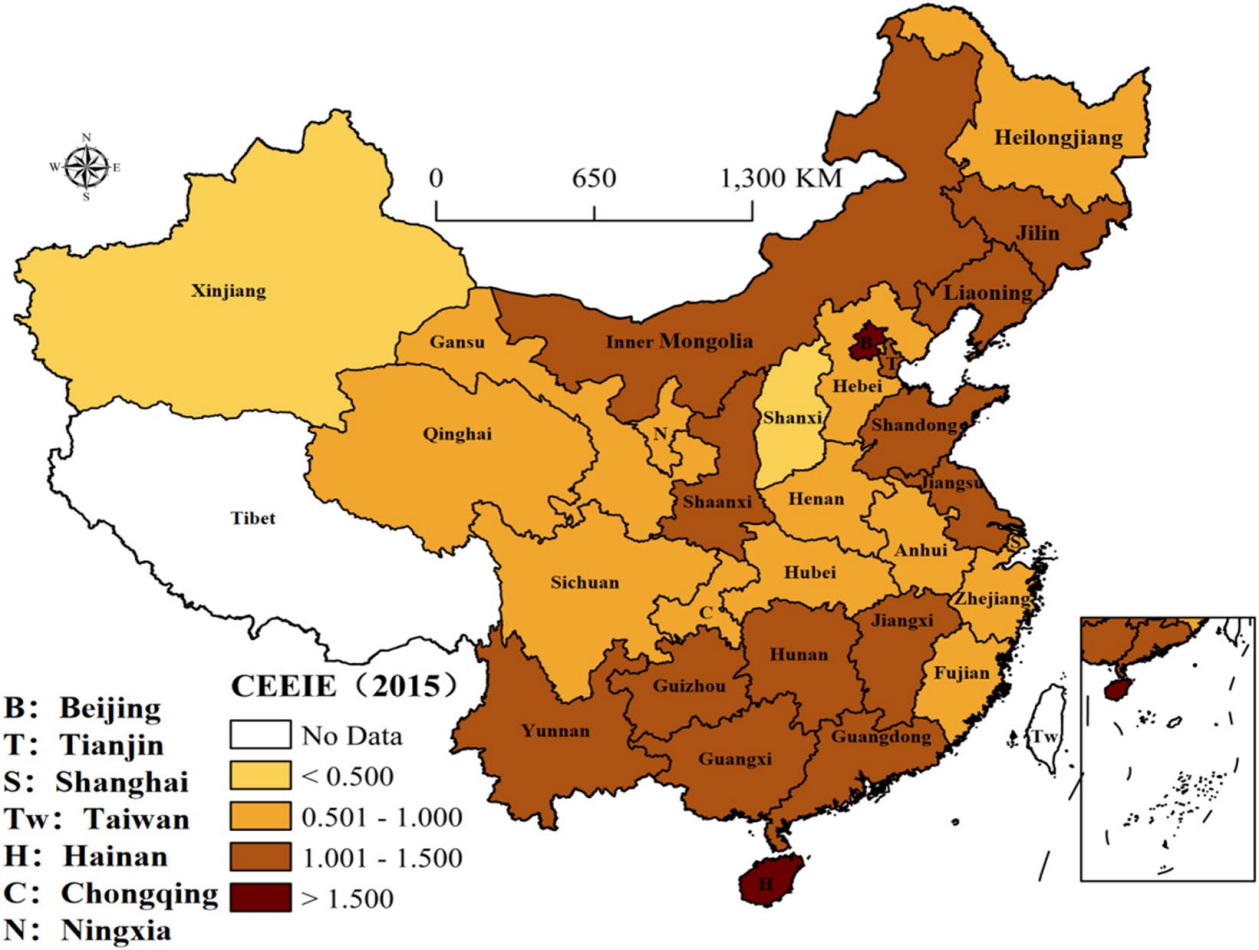
The distribution of CEEIE in 2015 (prepared by KL in ArcGIS Pro, https://www.esri.com/zh-cn/arcgis/products/arcgis-pro/resources).

**Figure 5:**
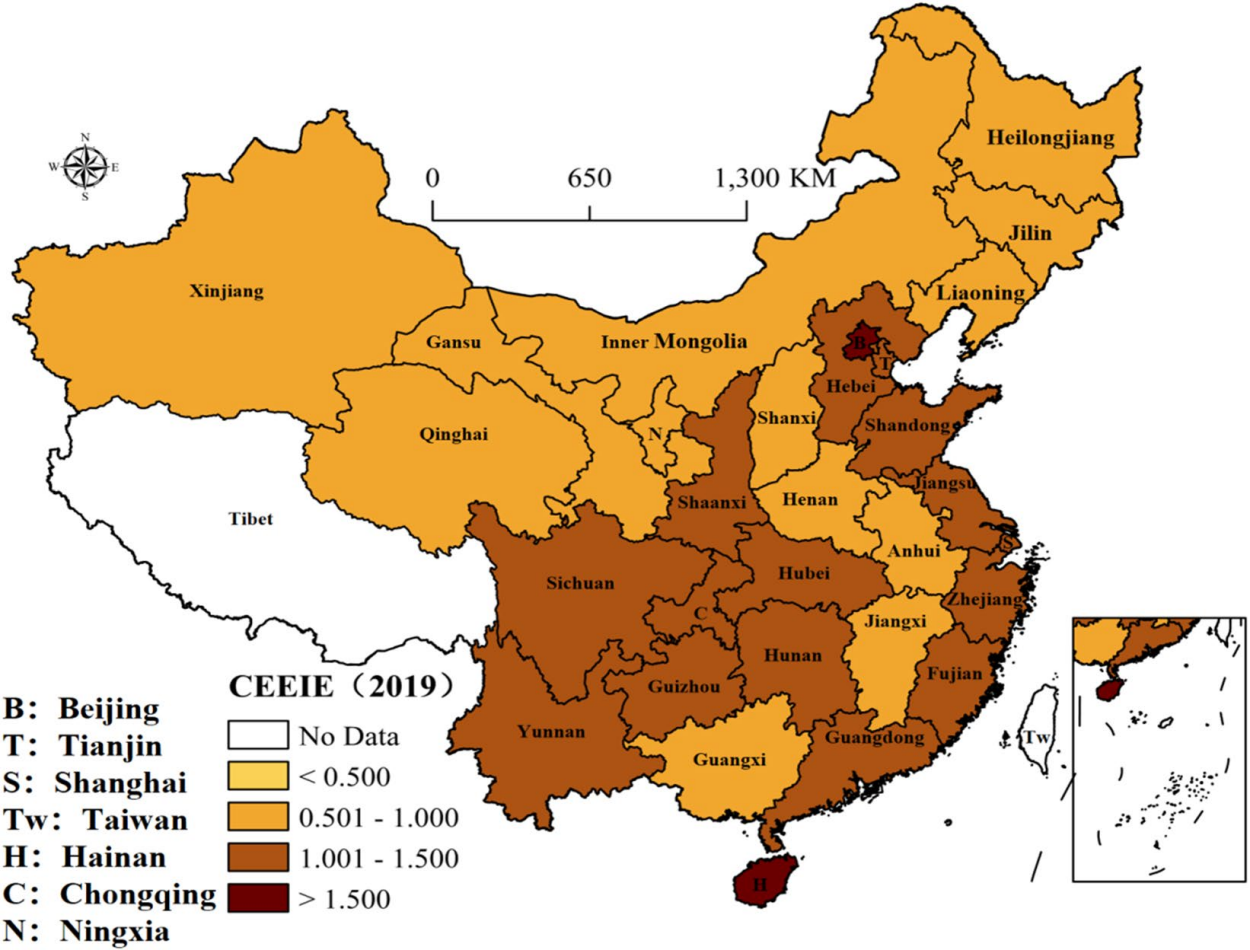
The distribution of CEEIE in 2019 (prepared by KL in ArcGIS Pro, https://www.esri.com/zh-cn/arcgis/products/arcgis-pro/resources).

As graphical supplements to CEEIE in Table 3, Figures 1, 2, 3, 4 and 5 illustrate the geographic distribution of CEEIE across China for 2000, 2005, 2010, 2015 and 2019. The CEEIE varies substantially across provinces and tends to cluster in space over time, which means that the spatial distribution of CEEIE is being from disorder to order. Specifically, provinces characterized by high CEEIE are shown to cluster in the coastal regions with developed economy or region attaching more importance to environment-friendly industries from 2000 to 2019. In China, developed provinces have transformed from deep industrialization to service-oriented industries and then realized sustainable emission reduction. For example, Beijing in northern coast region has been focusing on developing new energy utilization and cultivating industries related new energy, which also promotes the reduction of industrial carbon emissions. While Hunan in middle Yangtze River region has paid more attention to the environmental impact of the industry and tended to introduce low-carbon emission and environment-friendly industries.

### The regional difference of CEEIE

**Figure 6:**
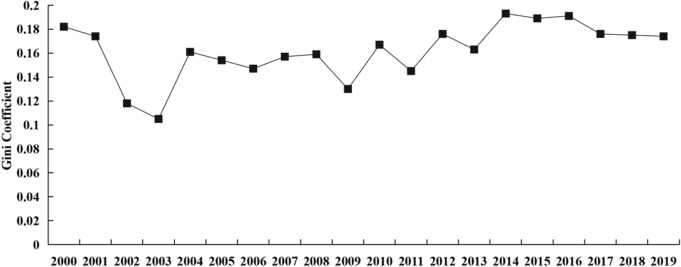
The total difference of CEEIE.

Figure 6 illustrates the variation of total Gini ratio of CEEIE in China from 2000 to 2019. The total Gini ratio ranges from 0.105 to 0.193, which means that there is a great change of CEEIE between provinces over time. Specifically, the highest total Gini ratio is 0.193 in 2014, the second highest is 0.191 in 2016, the lowest is 0.105 in 2003, but it is 0.176 in 2019. These results indicate that the total difference of CEEIE has a fluctuating and upward trend in the research period. As shown in Table 3, the provinces with high CEEIE also have high average annual growth rate, while the provinces with low CEEIE also have low average annual growth rate. Therefore, the gap of CEEIE among provinces in China is expanded over time.

The differences of CEEIE within region from 2000 to 2019 are reported in Table 4. We can find that the Gini ratio within region is increasing in northern coast and southern coast region, but deceasing in other six regions. From 2000 to 2019, the average annual growth rate of the Gini ratio of CEEIE within region is negative (− 0.61%). These results imply that the differences of CEEIE within region have been narrowed. We believe that the implementation of regional coordinated development strategy and the gap of economic development of provinces in a region are the two key factors affecting the Gini ratio within region. For example, eastern coast region has implemented regional integration development strategy of Yangtze River Delta since 2010 and economic development of Shanghai, Zhejiang and Jiangsu has always been very balanced, which make that eastern coast region has the lowest Gini ratio within region.

**Table 4:** The difference of CEEIE within region.

	Northern coast region	Eastern coast region	Southern coast region	Northeast region	Middle Yellow River region	Middle Yangtze River region	Southwest region	Northwest region	Average
2000	0.016	0.106	0.128	0.108	0.174	0.114	0.097	0.063	0.101
2001	0.049	0.056	0.152	0.110	0.155	0.133	0.113	0.132	0.113
2002	0.078	0.073	0.023	0.061	0.127	0.101	0.091	0.099	0.082
2003	0.055	0.017	0.013	0.092	0.107	0.107	0.093	0.105	0.074
2004	0.067	0.062	0.08	0.132	0.063	0.066	0.156	0.141	0.096
2005	0.061	0.083	0.119	0.143	0.129	0.103	0.089	0.132	0.107
2006	0.005	0.081	0.163	0.133	0.103	0.093	0.110	0.061	0.094
2007	0.009	0.084	0.199	0.131	0.103	0.091	0.111	0.034	0.095
2008	0.011	0.067	0.235	0.117	0.094	0.098	0.083	0.061	0.096
2009	0.011	0.056	0.144	0.047	0.117	0.087	0.053	0.107	0.078
2010	0.014	0.051	0.229	0.063	0.111	0.067	0.131	0.067	0.092
2011	0.036	0.018	0.189	0.077	0.119	0.082	0.110	0.094	0.091
2012	0.037	0.025	0.257	0.020	0.100	0.059	0.086	0.074	0.082
2013	0.06	0.034	0.204	0.014	0.13	0.062	0.043	0.077	0.078
2014	0.081	0.035	0.238	0.114	0.184	0.100	0.097	0.048	0.112
2015	0.134	0.039	0.26	0.099	0.181	0.077	0.068	0.039	0.112
2016	0.091	0.003	0.239	0.136	0.151	0.096	0.064	0.022	0.100
2017	0.120	0.029	0.198	0.098	0.114	0.069	0.064	0.035	0.091
2018	0.138	0.027	0.192	0.082	0.117	0.069	0.063	0.044	0.092
2019	0.138	0.025	0.185	0.077	0.111	0.072	0.071	0.039	0.09
Average	0.061	0.049	0.172	0.093	0.125	0.087	0.090	0.074	0.094
Average annual growth rate (%)	12.01	− 7.32	1.96	− 1.77	− 2.34	− 2.40	− 1.63	− 2.49	− 0.61

The differences of CEEIE between regions from 2000 to 2019 are reported in Table 5. There are maximum gap between southern coast region and northwest region, while the minimum gap emerges between northern coast region and eastern coast region. These findings are consistent with the difference of economic development between the two regions in China. For the variation of Gini ratio of CEEIE between regions over time, it shows that the differences of CEEIE between adjacent regions are decreasing but increasing between distant regions. For example, the lowest average annual growth rate of Gini ratio of CEEIE between regions (− 3.46%) is for eastern coast region and middle Yangtze River region, while the highest (5.63%) is for northern coast region and northwest region. Therefore, we can conclude that CEEIE may have spatial spillover effect and the regions with high CEEIE or the regions with low CEEIE may cluster in space, which is consistent with the results of spatial distribution analysis.

**Table 5:** The difference of CEEIE between regions.

	2000	2001	2002	2003	2004	2005	2006	2007	2008	2009	2010	2011	2012	2013	2014	2015	2016	2017	2018	2019	Average	Average annual growth rate (%)
1 and 2	0.077	0.090	0.084	0.045	0.068	0.074	0.048	0.049	0.046	0.037	0.035	0.035	0.046	0.077	0.076	0.112	0.067	0.100	0.108	0.108	0.069	1.80
1 and 3	0.220	0.237	0.124	0.067	0.106	0.113	0.112	0.138	0.163	0.099	0.163	0.131	0.185	0.151	0.183	0.227	0.201	0.170	0.177	0.176	0.157	− 1.17
1 and 4	0.070	0.087	0.089	0.084	0.121	0.123	0.099	0.099	0.102	0.110	0.087	0.092	0.113	0.136	0.134	0.137	0.161	0.174	0.18	0.179	0.119	5.07
1 and 5	0.139	0.126	0.113	0.094	0.133	0.102	0.061	0.061	0.058	0.071	0.069	0.088	0.075	0.112	0.157	0.176	0.149	0.180	0.185	0.179	0.116	1.34
1 and 6	0.086	0.115	0.107	0.096	0.119	0.115	0.085	0.085	0.094	0.077	0.050	0.075	0.059	0.083	0.134	0.121	0.121	0.125	0.132	0.128	0.100	2.12
1 and 7	0.094	0.100	0.101	0.079	0.133	0.123	0.075	0.084	0.083	0.072	0.119	0.129	0.128	0.130	0.130	0.111	0.096	0.115	0.116	0.117	0.107	1.16
1 and 8	0.076	0.103	0.095	0.087	0.134	0.129	0.128	0.141	0.137	0.107	0.154	0.146	0.177	0.185	0.215	0.218	0.206	0.228	0.22	0.215	0.155	5.63
2 and 3	0.199	0.181	0.098	0.032	0.100	0.125	0.154	0.182	0.208	0.131	0.195	0.148	0.214	0.177	0.199	0.228	0.207	0.163	0.159	0.158	0.163	− 1.21
2 and 4	0.110	0.096	0.073	0.082	0.126	0.137	0.131	0.130	0.114	0.108	0.091	0.073	0.083	0.078	0.092	0.078	0.108	0.103	0.108	0.105	0.101	− 0.25
2 and 5	0.156	0.123	0.110	0.094	0.137	0.119	0.100	0.098	0.089	0.100	0.092	0.085	0.078	0.101	0.138	0.139	0.109	0.117	0.119	0.113	0.111	− 1.68
2 and 6	0.119	0.107	0.095	0.099	0.122	0.126	0.108	0.103	0.098	0.083	0.065	0.061	0.048	0.054	0.092	0.064	0.072	0.061	0.063	0.061	0.085	− 3.46
2 and 7	0.104	0.100	0.090	0.072	0.136	0.129	0.107	0.109	0.084	0.069	0.122	0.113	0.100	0.071	0.092	0.062	0.047	0.056	0.055	0.057	0.089	− 3.12
2 and 8	0.140	0.129	0.104	0.081	0.139	0.143	0.136	0.141	0.125	0.114	0.152	0.128	0.151	0.132	0.165	0.153	0.149	0.157	0.150	0.142	0.137	0.08
3 and 4	0.209	0.223	0.079	0.098	0.175	0.186	0.210	0.233	0.258	0.184	0.238	0.191	0.264	0.225	0.249	0.255	0.296	0.236	0.243	0.243	0.215	0.80
3 and 5	0.216	0.237	0.116	0.112	0.203	0.161	0.166	0.186	0.205	0.150	0.207	0.177	0.227	0.206	0.262	0.281	0.273	0.238	0.242	0.236	0.205	0.47
3 and 6	0.223	0.226	0.100	0.117	0.189	0.177	0.182	0.198	0.232	0.153	0.198	0.171	0.212	0.176	0.236	0.230	0.248	0.183	0.186	0.178	0.191	− 1.18
3 and 7	0.181	0.216	0.094	0.090	0.179	0.181	0.172	0.196	0.208	0.135	0.247	0.203	0.256	0.198	0.223	0.211	0.214	0.167	0.165	0.161	0.185	− 0.61
3 and 8	0.284	0.259	0.144	0.089	0.188	0.193	0.220	0.248	0.266	0.180	0.289	0.222	0.317	0.267	0.308	0.331	0.326	0.282	0.279	0.275	0.248	− 0.17
4 and 5	0.158	0.142	0.104	0.105	0.111	0.147	0.134	0.134	0.130	0.148	0.128	0.114	0.120	0.128	0.165	0.154	0.152	0.110	0.107	0.103	0.130	− 2.23
4 and 6	0.120	0.129	0.088	0.104	0.108	0.127	0.120	0.118	0.112	0.094	0.091	0.090	0.089	0.087	0.110	0.090	0.126	0.100	0.099	0.106	0.105	− 0.65
4 and 7	0.107	0.113	0.083	0.102	0.151	0.125	0.130	0.127	0.105	0.076	0.113	0.105	0.069	0.039	0.108	0.084	0.111	0.096	0.100	0.110	0.103	0.15
4 and 8	0.130	0.132	0.108	0.108	0.141	0.143	0.108	0.100	0.100	0.090	0.097	0.105	0.081	0.075	0.137	0.152	0.104	0.100	0.082	0.075	0.108	− 2.85
5 and 6	0.161	0.146	0.117	0.109	0.069	0.130	0.116	0.117	0.118	0.123	0.098	0.105	0.085	0.105	0.155	0.140	0.131	0.110	0.113	0.114	0.118	− 1.80
5 and 7	0.142	0.136	0.110	0.106	0.139	0.128	0.111	0.115	0.107	0.112	0.149	0.135	0.130	0.114	0.148	0.131	0.115	0.109	0.113	0.116	0.123	− 1.06
5 and 8	0.175	0.153	0.126	0.114	0.114	0.142	0.140	0.150	0.145	0.144	0.171	0.146	0.169	0.160	0.180	0.174	0.146	0.110	0.105	0.102	0.143	− 2.80
6 and 7	0.114	0.125	0.098	0.107	0.135	0.103	0.111	0.109	0.093	0.071	0.120	0.116	0.106	0.082	0.101	0.074	0.083	0.070	0.069	0.076	0.098	− 2.11
6 and 8	0.132	0.143	0.119	0.117	0.114	0.123	0.100	0.107	0.104	0.109	0.143	0.126	0.152	0.137	0.136	0.157	0.137	0.141	0.131	0.138	0.128	0.23
7 and 8	0.152	0.131	0.116	0.103	0.154	0.116	0.130	0.130	0.107	0.094	0.134	0.110	0.108	0.085	0.140	0.156	0.142	0.144	0.138	0.145	0.127	− 0.25

1 is northern coast region; 2 is eastern coast region; 3 is southern coast region; 4 is northeast region; 5 is middle Yellow River region; 6 is middle Yangtze River region; 7 is southwest region; 8 is northwest region.

**Figure 7:**
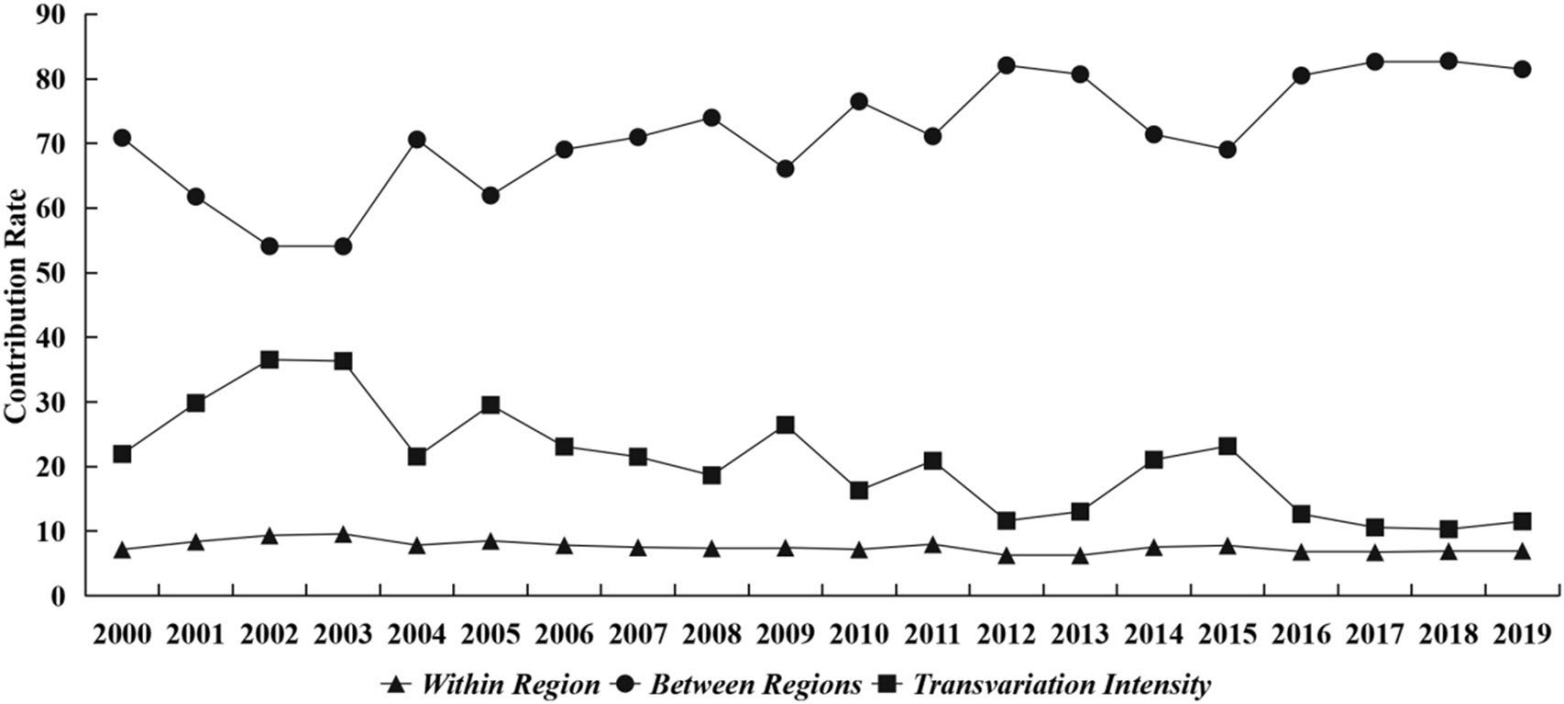
The trend of CR.

The sources of the regional difference of CEEIE are shown in Table 6 and Fig. 7. From 2000 to 2019, the Gini inequality within region contributes with a 7.58% to the total Gini ratio on average, the Gini inequality between regions contributes with a 71.59%, and the contribution of the transvariation intensity between regions is a 20.83%. As shown in Fig. 7, the contribution rate (CR) of the Gini inequality within region is stable, but the CR between regions is increasing and the CR of transvariation intensity is decreasing. These findings indicate that there are great differences between regions, which are the greatest sources of CEEIE inequality. Because the CR of transvariation intensity is the impact of the interaction of the difference within region and between regions on the total difference, its second largest CR means that the spatial dependence of the carbon emissions reduction behavior of local government also is an important source of the regional difference of CEEIE in China.

**Table 6:** The contributions to the total difference of CEEIE.

	Total difference	Difference within region		Difference between regions		Transvariation intensity between regions	
	*G*	*G*_*w*_	*CR* (%)	*G*_*rb*_	*CR* (%)	*G*_*t*_	*CR* (%)
2000	0.182	0.013	7.18	0.129	70.88	0.04	21.95
2001	0.174	0.015	8.40	0.108	61.77	0.052	29.83
2002	0.118	0.011	9.35	0.064	54.11	0.043	36.54
2003	0.105	0.01	9.58	0.057	54.09	0.038	36.33
2004	0.161	0.013	7.84	0.114	70.61	0.035	21.55
2005	0.154	0.013	8.52	0.095	61.95	0.045	29.53
2006	0.147	0.011	7.84	0.101	69.07	0.034	23.09
2007	0.157	0.012	7.51	0.111	70.98	0.034	21.51
2008	0.159	0.012	7.36	0.117	74.00	0.03	18.64
2009	0.13	0.01	7.46	0.086	66.09	0.034	26.45
2010	0.167	0.012	7.18	0.128	76.51	0.027	16.31
2011	0.145	0.012	7.99	0.103	71.12	0.03	20.90
2012	0.176	0.011	6.29	0.144	82.09	0.02	11.62
2013	0.163	0.01	6.27	0.132	80.70	0.021	13.03
2014	0.193	0.015	7.55	0.138	71.40	0.041	21.05
2015	0.189	0.015	7.79	0.131	69.053	0.044	23.16
2016	0.191	0.013	6.84	0.154	80.51	0.024	12.66
2017	0.176	0.012	6.76	0.145	82.65	0.019	10.59
2018	0.175	0.012	6.92	0.145	82.76	0.018	10.31
2019	0.174	0.012	6.99	0.141	81.48	0.02	11.5
Average	0.162	0.012	7.58	0.117	71.59	0.032	20.83
Average annual growth rate (%)	− 0.24	− 0.42	− 0.14	0.47	0.74	− 3.58	− 3.33

## Conclusions and implications

This paper uses the three-stage super-efficiency SBM-DEA model with undesirable output to evaluate CEEIE of 30 provinces in China from 2000 to 2017, and analyzes the spatial distribution and regional difference of CEEIE. We find that there is a big difference of CEEIE between provinces and between regions and the CEEIE of most provinces and regions are low. The analysis of spatial distribution indicates that the provinces characterized with high or low CEEIE cluster in space and the spatial distribution of provincial CEEIE is being from disorder to order over time. But the results of total Dagum Gini ratio indicate that the difference of CEEIE among provinces is expanded over time, which means that there are great gaps among the carbon emissions reduction capability of local government. Especially, the provinces with good economic foundation always have higher CEEIE. Furthermore, the decomposition of the total Dagum Gini ratio shows that the differences of CEEIE within region have been narrowed. Because the provinces in a same region have similar emissions reduction capability and it is more convenient for them to carry out carbon emission cooperation, the decreasing trend of the differences of CEEIE within region occurs. Besides, the differences between regions and its greatest contribution to the total difference of CEEIE imply that other than interprovincial carbon emission cooperation, interregional cooperation can play an important role in the regional difference of CEEIE. Finally, all the findings mean that the spatial dependence of carbon emissions reduction behavior of local government has a vital influence on CEEIE.

The findings of this study have some important policy implications for improving carbon emissions efficiency and promoting the balance of regional carbon emissions. First of all, the governments should implement differentiated schemes based on the actual situation of each region when formulating carbon emissions reduction policies. For example, the coast regions, as economic leading and industrial transfer out regions, have high degree of industrialization and economic development and strong demand for industrial upgrading and environmental improvement. Therefore, the governments in these regions should strengthen the current governance and intervention means to formulate more stringent carbon emissions reduction standards and promote the optimization of industrial structure and industrial carbon emissions. Nevertheless, the economic zones located in the center and west (such as middle Yellow River region) have relatively low level of economic development, and they are also the main areas for undertaking industrial transfer. They thus bear the dual pressures of economic development and environmental improvement. The governments in these regions should play better roles in supervision and guidance, and take into account the obligation of carbon emissions reduction when pursuing economic growth. For example, formulating scientific industrial development plans based on regional characteristics and functional positioning, and effectively screening the transferred industries through reasonable guidance and intervention may avoid the possible negative effects of industrial transfer on environmental pollution in the process of economic development.

Second, the interregional integration policies for carbon emissions reduction should be made by central government to enhance the CEEIE. Most importantly, it is necessary to establish an effective interregional cooperative mechanism to draw up a long-range carbon emissions reduction plan. For example, it is practicable to establish an integrated management organization for interregional environmental management, or establish an interregional environmental protection organization. The agency should formulate a clear action plan for interregional integration of carbon emissions reduction policy, which is not only a comprehensive plan, but must be implemented into environmental protection policy of each province in different regions.

Third, the provinces in China should regularly share energy saving and emissions reduction technology and exchange the carbon emissions reduction policies. Green technology innovation activities are mainly concentrated in the developed regions of China, in which there are better capitals and technology endowment advantages. Therefore, the realization of high-efficiency carbon emissions reduction depends on the deep integration of technical endowment between the developed regions and the underdeveloped regions. The conventional sharing of the experiences of carbon emissions reduction can make green technologies spread rapidly, which should give fully positive role for spillover effects of science and technology of industrial carbon emissions reduction.
